# *N*-(3,5-Dimethyl­phen­yl)-2-nitro­benzene­sulfonamide

**DOI:** 10.1107/S1600536812036926

**Published:** 2012-09-01

**Authors:** U. Chaithanya, Sabine Foro, B. Thimme Gowda

**Affiliations:** a Department of Chemistry, Mangalore University, Mangalagangotri 574 199, Mangalore, India; b Institute of Materials Science, Darmstadt University of Technology, Petersenstrasse 23, D-64287 Darmstadt, Germany

## Abstract

The asymmetric unit of the title compound, C_14_H_14_N_2_O_4_S, consists of two crystallographically independent mol­ecules. The mol­ecules are twisted at the S—N bonds with C—S—N—C torsion angles of 44.2 (3) and −49.3 (3)°. The dihedral angles between the benzene rings in the two mol­ecules are 71.53 (7) and 72.11 (7)°. The amide H atoms exhibit bifurcated intra- and inter­molecular hydrogen bonds; the intra­molecular N—H⋯O(N) hydrogen bonds generate *S*(7) motifs. In the crystal, the independent mol­ecules are separately connected through the inter­molecular N—H⋯O(S) hydrogen bonds, generating a *C*(4) motif and a helical chain along the *b* axis for one mol­ecule and an *R*_2_^2^(8) motif and an inversion dimer for the other. The crystal studied was a pseudo-merohedral twin with twin law (-100/0-10/001), the refined ratio of the twin domains being 0.7876 (12):0.2124 (12).

## Related literature

For studies on the effects of substituents on the structures and other aspects of *N*-(ar­yl)-amides, see: Gowda & Weiss (1994 ▶); Shahwar *et al.* (2012 ▶), of *N*-aryl­sulfonamides, see: Chaithanya *et al.* (2012 ▶) and of *N*-chloro­aryl­sulfonamides, see: Shetty & Gowda (2004 ▶). For hydrogen-bonding patterns and motifs, see: Adsmond *et al.* (2001 ▶),
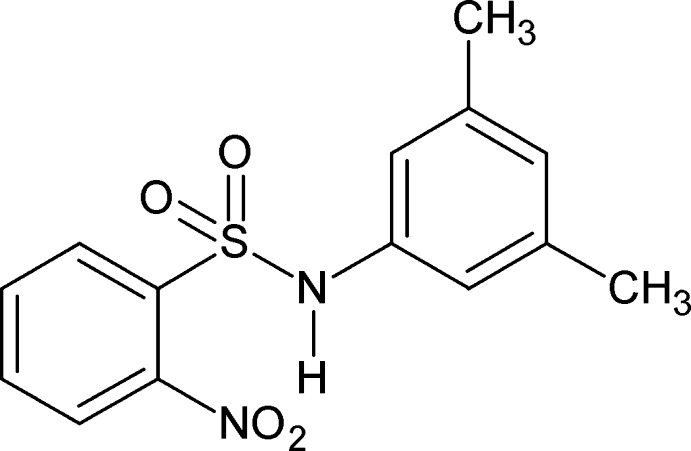


## Experimental

### 

#### Crystal data

C_14_H_14_N_2_O_4_S
*M* *_r_* = 306.33
Monoclinic, 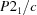

*a* = 16.561 (1) Å
*b* = 8.1611 (6) Å
*c* = 21.476 (2) Å
β = 90.056 (7)°
*V* = 2902.6 (4) Å^3^
*Z* = 8
Mo *K*α radiation
μ = 0.24 mm^−1^
*T* = 293 K
0.48 × 0.40 × 0.20 mm

#### Data collection

Oxford Diffraction Xcalibur diffractometer with a Sapphire CCD detector
Absorption correction: multi-scan (*CrysAlis RED*; Oxford Diffraction, 2009 ▶) *T*_min_ = 0.894, *T*_max_ = 0.954
12941 measured reflections
5929 independent reflections
4004 reflections with *I* > 2σ(*I*)
*R*_int_ = 0.030

#### Refinement

*R*[*F* ^2^ > 2σ(*F* ^2^)] = 0.047
*wR*(*F* ^2^) = 0.123
*S* = 1.00
5929 reflections
390 parameters
6 restraints
H atoms treated by a mixture of independent and constrained refinement
Δρ_max_ = 0.32 e Å^−3^
Δρ_min_ = −0.29 e Å^−3^

### 

Data collection: *CrysAlis CCD* (Oxford Diffraction, 2009 ▶); cell refinement: *CrysAlis CCD*; data reduction: *CrysAlis RED* (Oxford Diffraction, 2009 ▶); program(s) used to solve structure: *SHELXS97* (Sheldrick, 2008 ▶); program(s) used to refine structure: *SHELXL97* (Sheldrick, 2008 ▶); molecular graphics: *PLATON* (Spek, 2009 ▶); software used to prepare material for publication: *SHELXL97*.

## Supplementary Material

Crystal structure: contains datablock(s) I, global. DOI: 10.1107/S1600536812036926/is5184sup1.cif

Structure factors: contains datablock(s) I. DOI: 10.1107/S1600536812036926/is5184Isup2.hkl

Supplementary material file. DOI: 10.1107/S1600536812036926/is5184Isup3.cml

Additional supplementary materials:  crystallographic information; 3D view; checkCIF report

## Figures and Tables

**Table 1:** Hydrogen-bond geometry (Å, °)

*D*—H⋯*A*	*D*—H	H⋯*A*	*D*⋯*A*	*D*—H⋯*A*
N1—H1*N*⋯O1^i^	0.82 (2)	2.40 (2)	3.089 (3)	142 (3)
N1—H1*N*⋯O3	0.82 (2)	2.52 (3)	2.893 (4)	109 (2)
N3—H3*N*⋯O7	0.83 (2)	2.42 (3)	2.963 (3)	124 (3)
N3—H3*N*⋯O6^ii^	0.83 (2)	2.54 (2)	3.195 (3)	136 (3)

Symmetry codes: (i) 
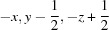
; (ii) 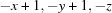.

**Fig. 1.**
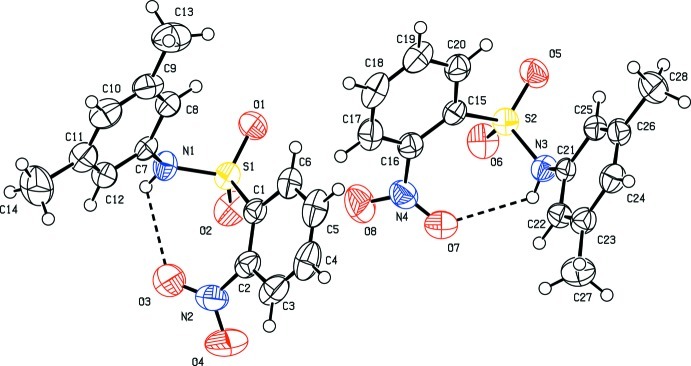
The asymmetric unit of the title compound, showing the atom labelling scheme and with displacement ellipsoids drawn at the 50% probability level. The intramolecular N—H···O hydrogen bonds are shown as dashed lines.

**Fig. 2.**
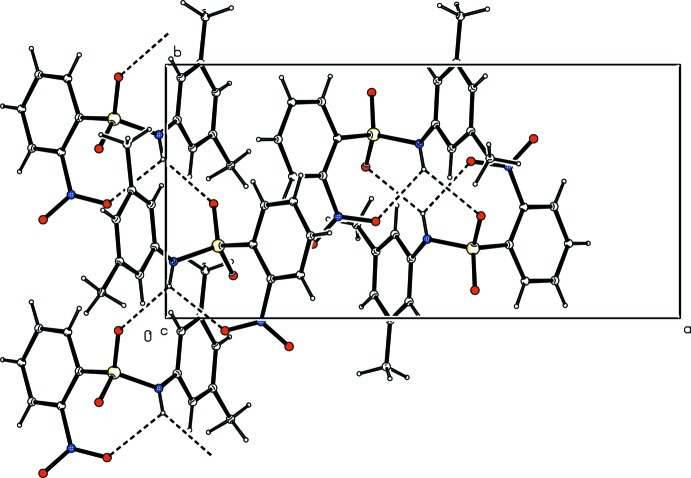
A molecular packing diagram of the title compound with the N—H···O hydrogen bonds shown as dashed lines.

## supplementary crystallographic information

### Comment

As a part of our studies on the substituent effects on the structures and other aspects of *N*-(aryl)-amides (Gowda & Weiss, 1994; Shahwar *et al.*, 2012), *N*-arylsulfonamides (Chaithanya *et al.*, 2012) and *N*-chloroarylsulfonamides (Shetty & Gowda, 2004), in the present work, the crystal structure of *N*-(3,5-dimethylphenyl)-2-nitrobenzenesulfonamide has been determined.

The asymmetric unit of the title compound consists of two crystallographically independent molecules (Fig. 1). The conformation of the N—C bond in the —SO_2_—NH—C segment has *gauche* torsions with respect to the S═O bonds, similar to that observed in *N*-(3-methylphenyl)-2-nitrobenzenesulfonamide (I) (Chaithanya *et al.*, 2012). Further, the conformation of the N—H bond in the —SO_2_—NH— segment is *syn* to the *ortho*-nitro group in the sulfonyl benzene ring. The molecules are twisted at the S—N bonds with C1—S1—N1—C7 and C15—S2—N3—C21 torsion angles of 44.2 (3) and -49.3 (3)°, respectively, compared to the value of 46.97 (16)° in (I). The dihedral angles between the sulfonyl and the anilino rings in the two molecules are 71.53 (7) and 72.11 (7)°, compared to the value of 73.64 (7)° in (I). In each molecule the amide H atom shows an intramolecular hydrogen bond (N1—H1N···O1 and N3—H3N···O7; Table 1) with the O atom of the *ortho*-nitro group in the sulfonyl benzene ring, generating an S(7) motif (Adsmond *et al.*, 2001). In the crystal, the amide H atoms show intermolecular hydrogen bonds with the sulfonyl oxygen atoms of the other molecule; the N1—H1N···O1^i^ hydrogen bond (symmetry code in Table 1) generates a C(4) motif and a helical chain along the *b* axis, while the N3—H3N···O6^ii^ hydrogen bond (symmetry code in Table 1) an R^2^_2_(8) motif and an inversion dimer. A part of the crystal structure is shown in Fig. 2.

### Experimental

The title compound was prepared by treating 2-nitrobenzenesulfonylchloride with 3,5-dimethylaniline in the stoichiometric ratio and boiling the reaction mixture for 15 minutes. The reaction mixture was then cooled to room temperature and added to ice cold water (100 ml). The resultant solid, *N*-(3,5-dimethylphenyl)-2-nitrobenzenesulfonamide was filtered under suction and washed thoroughly with cold water and dilute HCl to remove the excess sulfonylchloride and aniline, respectively. It was then recrystallized to constant melting point from dilute ethanol. The purity of the compound was checked and characterized by its infrared spectra. Prism like brown single crystals of the title compound suitable for X-ray diffraction studies were grown in an ethanolic solution by slow evaporation of the solvent at room temperature.

### Refinement

H atoms bonded to C were positioned with idealized geometry using a riding model with the aromatic C—H = 0.93 Å and the methyl C—H = 0.96 Å. The positions of amino H atoms were refined with the N—H distance restrained to 0.86 (2) Å. All H atoms were refined with isotropic displacement parameters set at 1.2*U*_eq_(C-aromatic, N) and 1.5*U*_eq_ (*C*-methyl). Rigid-bond restraints (*DELU*) were applied for atom paris of C18/C19, N2/C2, O3/N2 and N4/C16. The crystal was refined with the twin law (-1 0 0/0 -1 0/0 0 1).

### Crystal data

**Table:**

C_14_H_14_N_2_O_4_S	*F*(000) = 1280
*M**_r_* = 306.33	*D*_x_ = 1.402 Mg m^−^^3^
Monoclinic, *P*2_1_/*c*	Mo *K*α radiation, λ = 0.71073 Å
Hall symbol: -P 2ybc	Cell parameters from 2953 reflections
*a* = 16.561 (1) Å	θ = 2.5–27.9°
*b* = 8.1611 (6) Å	µ = 0.24 mm^−^^1^
*c* = 21.476 (2) Å	*T* = 293 K
β = 90.056 (7)°	Prism, brown
*V* = 2902.6 (4) Å^3^	0.48 × 0.40 × 0.20 mm
*Z* = 8	

### Data collection

**Table:**

Oxford Diffraction Xcalibur diffractometer with a Sapphire CCD detector	5929 independent reflections
Radiation source: fine-focus sealed tube	4004 reflections with *I* > 2σ(*I*)
Graphite monochromator	*R*_int_ = 0.030
ω scans	θ_max_ = 26.4°, θ_min_ = 2.5°
Absorption correction: multi-scan (*CrysAlis RED*; Oxford Diffraction, 2009)	*h* = −20→20
*T*_min_ = 0.894, *T*_max_ = 0.954	*k* = −10→5
12941 measured reflections	*l* = −19→26

### Refinement

**Table:**

Refinement on *F*^2^	Primary atom site location: structure-invariant direct methods
Least-squares matrix: full	Secondary atom site location: difference Fourier map
*R*[*F*^2^ > 2σ(*F*^2^)] = 0.047	Hydrogen site location: inferred from neighbouring sites
*wR*(*F*^2^) = 0.123	H atoms treated by a mixture of independent and constrained refinement
*S* = 1.00	*w* = 1/[σ^2^(*F*_o_^2^) + (0.0703*P*)^2^] where *P* = (*F*_o_^2^ + 2*F*_c_^2^)/3
5929 reflections	(Δ/σ)_max_ = 0.006
390 parameters	Δρ_max_ = 0.32 e Å^−^^3^
6 restraints	Δρ_min_ = −0.29 e Å^−^^3^

### Special details

**Table:**

Geometry. All e.s.d.'s (except the e.s.d. in the dihedral angle between two l.s. planes) are estimated using the full covariance matrix. The cell e.s.d.'s are taken into account individually in the estimation of e.s.d.'s in distances, angles and torsion angles; correlations between e.s.d.'s in cell parameters are only used when they are defined by crystal symmetry. An approximate (isotropic) treatment of cell e.s.d.'s is used for estimating e.s.d.'s involving l.s. planes.
Refinement. Refinement of *F*^2^ against ALL reflections. The weighted *R*-factor *wR* and goodness of fit *S* are based on *F*^2^, conventional *R*-factors *R* are based on *F*, with *F* set to zero for negative *F*^2^. The threshold expression of *F*^2^ > σ(*F*^2^) is used only for calculating *R*-factors(gt) *etc*. and is not relevant to the choice of reflections for refinement. *R*-factors based on *F*^2^ are statistically about twice as large as those based on *F*, and *R*- factors based on ALL data will be even larger.

### Fractional atomic coordinates and isotropic or equivalent isotropic displacement parameters (Å^2^)

**Table:**

	*x*	*y*	*z*	*U*_iso_*/*U*_eq_	
S1	0.10145 (4)	0.28567 (9)	0.27572 (3)	0.0436 (2)	
O1	0.09244 (13)	0.4509 (3)	0.25622 (10)	0.0612 (6)	
O2	0.13065 (14)	0.1683 (3)	0.23195 (10)	0.0648 (6)	
O3	0.11511 (16)	−0.0493 (3)	0.33802 (14)	0.0830 (8)	
O4	0.23948 (19)	−0.1115 (4)	0.34483 (18)	0.1090 (11)	
N1	0.01449 (14)	0.2232 (3)	0.30039 (11)	0.0451 (6)	
H1N	0.0052 (18)	0.127 (2)	0.2925 (14)	0.054*	
N2	0.18483 (18)	−0.0130 (3)	0.34824 (13)	0.0597 (7)	
C1	0.16894 (15)	0.2911 (3)	0.34040 (13)	0.0390 (6)	
C2	0.20431 (17)	0.1548 (4)	0.36690 (13)	0.0459 (7)	
C3	0.26122 (18)	0.1709 (5)	0.41434 (16)	0.0614 (9)	
H3	0.2854	0.0785	0.4315	0.074*	
C4	0.2813 (2)	0.3250 (6)	0.43555 (16)	0.0657 (10)	
H4	0.3182	0.3364	0.4680	0.079*	
C5	0.24775 (18)	0.4600 (5)	0.40949 (17)	0.0634 (10)	
H5	0.2625	0.5635	0.4236	0.076*	
C6	0.19187 (17)	0.4449 (4)	0.36223 (14)	0.0513 (8)	
H6	0.1692	0.5384	0.3447	0.062*	
C7	−0.02146 (14)	0.2819 (3)	0.35635 (13)	0.0374 (6)	
C8	−0.02904 (17)	0.4483 (4)	0.36658 (14)	0.0454 (7)	
H8	−0.0092	0.5225	0.3375	0.054*	
C9	−0.06662 (18)	0.5047 (4)	0.42075 (16)	0.0542 (8)	
C10	−0.0970 (2)	0.3898 (4)	0.46210 (15)	0.0592 (9)	
H10	−0.1228	0.4262	0.4980	0.071*	
C11	−0.09039 (19)	0.2245 (4)	0.45202 (15)	0.0538 (8)	
C12	−0.05146 (17)	0.1705 (4)	0.39875 (14)	0.0467 (7)	
H12	−0.0455	0.0588	0.3915	0.056*	
C13	−0.0759 (2)	0.6871 (4)	0.4317 (2)	0.0811 (12)	
H13A	−0.0327	0.7445	0.4114	0.122*	
H13B	−0.0741	0.7091	0.4756	0.122*	
H13C	−0.1267	0.7233	0.4151	0.122*	
C14	−0.1251 (3)	0.1020 (5)	0.49760 (18)	0.0875 (13)	
H14A	−0.0820	0.0517	0.5206	0.131*	
H14B	−0.1545	0.0194	0.4753	0.131*	
H14C	−0.1608	0.1572	0.5258	0.131*	
S2	0.40323 (4)	0.72449 (9)	0.02986 (3)	0.04244 (19)	
O5	0.39955 (14)	0.8897 (2)	0.01021 (10)	0.0578 (6)	
O6	0.38793 (14)	0.5967 (3)	−0.01392 (9)	0.0610 (6)	
O7	0.40738 (16)	0.3814 (3)	0.09201 (14)	0.0767 (7)	
O8	0.28562 (16)	0.2925 (3)	0.08814 (15)	0.0886 (9)	
N3	0.49231 (14)	0.6879 (3)	0.05761 (11)	0.0437 (6)	
H3N	0.5007 (18)	0.588 (2)	0.0555 (14)	0.052*	
N4	0.33469 (18)	0.4009 (3)	0.09653 (12)	0.0542 (6)	
C15	0.33333 (15)	0.7097 (3)	0.09236 (13)	0.0386 (6)	
C16	0.30457 (16)	0.5622 (4)	0.11719 (13)	0.0439 (7)	
C17	0.24623 (18)	0.5593 (5)	0.16329 (15)	0.0552 (8)	
H17	0.2266	0.4602	0.1784	0.066*	
C18	0.21766 (18)	0.7054 (5)	0.18641 (17)	0.0616 (9)	
H18	0.1798	0.7049	0.2183	0.074*	
C19	0.24443 (18)	0.8513 (5)	0.16295 (16)	0.0593 (8)	
H19	0.2243	0.9492	0.1787	0.071*	
C20	0.30193 (17)	0.8538 (4)	0.11534 (14)	0.0489 (8)	
H20	0.3191	0.9535	0.0991	0.059*	
C21	0.52374 (15)	0.7656 (3)	0.11202 (12)	0.0343 (6)	
C22	0.55543 (16)	0.6691 (4)	0.15836 (14)	0.0431 (7)	
H22	0.5518	0.5557	0.1551	0.052*	
C23	0.59271 (17)	0.7376 (4)	0.20976 (14)	0.0457 (7)	
C24	0.59616 (17)	0.9071 (4)	0.21341 (14)	0.0486 (7)	
H24	0.6207	0.9549	0.2479	0.058*	
C25	0.56453 (16)	1.0072 (4)	0.16781 (15)	0.0452 (7)	
C26	0.52802 (16)	0.9346 (4)	0.11635 (13)	0.0418 (7)	
H26	0.5065	0.9996	0.0849	0.050*	
C27	0.6301 (2)	0.6305 (4)	0.25904 (16)	0.0674 (10)	
H27A	0.6015	0.5284	0.2611	0.101*	
H27B	0.6271	0.6847	0.2986	0.101*	
H27C	0.6856	0.6101	0.2488	0.101*	
C28	0.5723 (2)	1.1911 (4)	0.17188 (19)	0.0668 (10)	
H28A	0.5320	1.2333	0.1996	0.100*	
H28B	0.5650	1.2380	0.1313	0.100*	
H28C	0.6250	1.2189	0.1873	0.100*	

### Atomic displacement parameters (Å^2^)

**Table:**

	*U*^11^	*U*^22^	*U*^33^	*U*^12^	*U*^13^	*U*^23^
S1	0.0409 (4)	0.0553 (5)	0.0346 (4)	−0.0001 (3)	−0.0008 (3)	0.0018 (3)
O1	0.0596 (13)	0.0618 (14)	0.0622 (14)	−0.0008 (11)	0.0025 (11)	0.0275 (12)
O2	0.0650 (14)	0.0870 (17)	0.0424 (12)	0.0065 (12)	0.0043 (11)	−0.0184 (12)
O3	0.0751 (14)	0.0599 (15)	0.114 (2)	−0.0024 (13)	−0.0071 (16)	−0.0043 (15)
O4	0.096 (2)	0.0755 (19)	0.156 (3)	0.0428 (17)	0.013 (2)	0.0038 (19)
N1	0.0412 (13)	0.0502 (15)	0.0438 (14)	−0.0084 (12)	−0.0014 (11)	−0.0094 (13)
N2	0.0642 (14)	0.0532 (13)	0.0617 (17)	0.0132 (13)	0.0073 (14)	0.0081 (13)
C1	0.0330 (14)	0.0462 (17)	0.0378 (14)	0.0001 (13)	0.0039 (12)	0.0016 (14)
C2	0.0381 (15)	0.0598 (15)	0.0399 (15)	0.0035 (13)	0.0060 (13)	0.0067 (14)
C3	0.0425 (18)	0.092 (3)	0.0492 (19)	0.0106 (18)	0.0040 (15)	0.019 (2)
C4	0.0446 (18)	0.109 (3)	0.0433 (18)	−0.014 (2)	−0.0004 (15)	−0.004 (2)
C5	0.0468 (19)	0.085 (3)	0.058 (2)	−0.0163 (18)	0.0061 (17)	−0.020 (2)
C6	0.0425 (16)	0.058 (2)	0.0534 (18)	−0.0078 (15)	0.0032 (14)	−0.0068 (16)
C7	0.0261 (13)	0.0455 (17)	0.0407 (15)	0.0015 (12)	−0.0052 (11)	−0.0030 (13)
C8	0.0401 (16)	0.0463 (19)	0.0497 (17)	−0.0005 (13)	−0.0039 (14)	0.0021 (14)
C9	0.0513 (18)	0.0493 (19)	0.062 (2)	0.0106 (15)	−0.0108 (16)	−0.0082 (17)
C10	0.058 (2)	0.072 (2)	0.0484 (18)	0.0116 (18)	0.0051 (17)	−0.0093 (17)
C11	0.0498 (18)	0.067 (2)	0.0446 (17)	0.0013 (17)	0.0011 (15)	0.0028 (16)
C12	0.0428 (16)	0.0439 (18)	0.0535 (18)	−0.0011 (13)	−0.0032 (14)	0.0026 (15)
C13	0.088 (3)	0.062 (3)	0.093 (3)	0.020 (2)	−0.008 (2)	−0.017 (2)
C14	0.097 (3)	0.099 (3)	0.067 (2)	−0.014 (2)	0.020 (2)	0.020 (2)
S2	0.0462 (4)	0.0507 (4)	0.0305 (3)	−0.0034 (4)	−0.0045 (3)	0.0018 (3)
O5	0.0634 (14)	0.0569 (13)	0.0531 (12)	−0.0055 (11)	−0.0033 (11)	0.0177 (10)
O6	0.0708 (15)	0.0708 (15)	0.0413 (11)	−0.0095 (12)	−0.0052 (11)	−0.0146 (11)
O7	0.0674 (16)	0.0550 (15)	0.108 (2)	0.0121 (12)	0.0070 (15)	0.0038 (14)
O8	0.0862 (19)	0.0584 (16)	0.121 (2)	−0.0242 (14)	0.0008 (18)	−0.0154 (16)
N3	0.0415 (13)	0.0444 (15)	0.0452 (14)	0.0019 (12)	0.0028 (11)	−0.0079 (12)
N4	0.0623 (17)	0.0468 (14)	0.0534 (16)	−0.0029 (13)	−0.0035 (14)	0.0078 (12)
C15	0.0324 (14)	0.0463 (17)	0.0369 (15)	−0.0008 (13)	−0.0066 (12)	−0.0018 (13)
C16	0.0399 (15)	0.0520 (16)	0.0397 (16)	−0.0037 (13)	−0.0073 (13)	0.0024 (13)
C17	0.0470 (18)	0.068 (2)	0.0505 (19)	−0.0107 (16)	0.0006 (15)	0.0065 (17)
C18	0.0378 (16)	0.094 (2)	0.0528 (19)	−0.0051 (17)	0.0027 (15)	−0.0098 (18)
C19	0.0414 (17)	0.0705 (19)	0.066 (2)	0.0048 (14)	−0.0040 (16)	−0.0244 (17)
C20	0.0416 (16)	0.0505 (19)	0.0546 (18)	−0.0029 (14)	−0.0067 (15)	−0.0046 (15)
C21	0.0290 (13)	0.0364 (16)	0.0376 (14)	−0.0023 (11)	0.0051 (11)	0.0011 (12)
C22	0.0425 (16)	0.0376 (16)	0.0494 (17)	−0.0002 (12)	0.0025 (13)	−0.0015 (14)
C23	0.0410 (15)	0.0537 (19)	0.0424 (16)	0.0004 (14)	−0.0013 (14)	0.0041 (14)
C24	0.0442 (16)	0.0553 (19)	0.0464 (17)	−0.0043 (14)	−0.0043 (14)	−0.0058 (15)
C25	0.0410 (16)	0.0407 (17)	0.0540 (18)	−0.0055 (13)	0.0034 (14)	−0.0028 (14)
C26	0.0359 (14)	0.0437 (18)	0.0458 (16)	−0.0021 (12)	0.0015 (13)	0.0070 (14)
C27	0.071 (2)	0.073 (2)	0.058 (2)	0.0065 (19)	−0.0150 (18)	0.0135 (18)
C28	0.072 (2)	0.046 (2)	0.083 (3)	−0.0094 (16)	−0.002 (2)	−0.0081 (18)

### Geometric parameters (Å, º)

**Table:**

S1—O1	1.420 (2)	S2—O5	1.414 (2)
S1—O2	1.427 (2)	S2—O6	1.427 (2)
S1—N1	1.617 (2)	S2—N3	1.618 (2)
S1—C1	1.783 (3)	S2—C15	1.777 (3)
O3—N2	1.212 (3)	O7—N4	1.218 (3)
O4—N2	1.213 (3)	O8—N4	1.215 (3)
N1—C7	1.425 (3)	N3—C21	1.427 (3)
N1—H1N	0.819 (17)	N3—H3N	0.832 (17)
N2—C2	1.463 (4)	N4—C16	1.476 (4)
C1—C2	1.379 (4)	C15—C20	1.378 (4)
C1—C6	1.392 (4)	C15—C16	1.401 (4)
C2—C3	1.393 (4)	C16—C17	1.384 (4)
C3—C4	1.378 (5)	C17—C18	1.375 (5)
C3—H3	0.9300	C17—H17	0.9300
C4—C5	1.355 (5)	C18—C19	1.367 (5)
C4—H4	0.9300	C18—H18	0.9300
C5—C6	1.378 (4)	C19—C20	1.398 (5)
C5—H5	0.9300	C19—H19	0.9300
C6—H6	0.9300	C20—H20	0.9300
C7—C12	1.380 (4)	C21—C22	1.373 (4)
C7—C8	1.381 (4)	C21—C26	1.384 (4)
C8—C9	1.398 (4)	C22—C23	1.382 (4)
C8—H8	0.9300	C22—H22	0.9300
C9—C10	1.386 (5)	C23—C24	1.386 (4)
C9—C13	1.515 (4)	C23—C27	1.506 (4)
C10—C11	1.370 (5)	C24—C25	1.378 (4)
C10—H10	0.9300	C24—H24	0.9300
C11—C12	1.386 (4)	C25—C26	1.392 (4)
C11—C14	1.513 (5)	C25—C28	1.509 (4)
C12—H12	0.9300	C26—H26	0.9300
C13—H13A	0.9600	C27—H27A	0.9600
C13—H13B	0.9600	C27—H27B	0.9600
C13—H13C	0.9600	C27—H27C	0.9600
C14—H14A	0.9600	C28—H28A	0.9600
C14—H14B	0.9600	C28—H28B	0.9600
C14—H14C	0.9600	C28—H28C	0.9600
			
O1—S1—O2	118.60 (14)	O5—S2—O6	119.54 (13)
O1—S1—N1	107.63 (13)	O5—S2—N3	108.96 (13)
O2—S1—N1	107.85 (14)	O6—S2—N3	105.60 (13)
O1—S1—C1	105.78 (13)	O5—S2—C15	105.19 (13)
O2—S1—C1	108.50 (13)	O6—S2—C15	109.43 (13)
N1—S1—C1	108.08 (12)	N3—S2—C15	107.66 (12)
C7—N1—S1	122.89 (19)	C21—N3—S2	123.45 (19)
C7—N1—H1N	115 (2)	C21—N3—H3N	115 (2)
S1—N1—H1N	114 (2)	S2—N3—H3N	108 (2)
O3—N2—O4	122.5 (3)	O8—N4—O7	123.6 (3)
O3—N2—C2	119.2 (3)	O8—N4—C16	117.9 (3)
O4—N2—C2	118.2 (3)	O7—N4—C16	118.3 (3)
C2—C1—C6	118.2 (3)	C20—C15—C16	118.0 (3)
C2—C1—S1	124.6 (2)	C20—C15—S2	117.3 (2)
C6—C1—S1	117.1 (2)	C16—C15—S2	124.6 (2)
C1—C2—C3	120.8 (3)	C17—C16—C15	121.6 (3)
C1—C2—N2	123.2 (3)	C17—C16—N4	115.9 (3)
C3—C2—N2	115.9 (3)	C15—C16—N4	122.5 (3)
C4—C3—C2	119.4 (3)	C18—C17—C16	119.0 (3)
C4—C3—H3	120.3	C18—C17—H17	120.5
C2—C3—H3	120.3	C16—C17—H17	120.5
C5—C4—C3	120.4 (3)	C19—C18—C17	120.7 (3)
C5—C4—H4	119.8	C19—C18—H18	119.7
C3—C4—H4	119.8	C17—C18—H18	119.7
C4—C5—C6	120.4 (3)	C18—C19—C20	120.3 (3)
C4—C5—H5	119.8	C18—C19—H19	119.9
C6—C5—H5	119.8	C20—C19—H19	119.9
C5—C6—C1	120.8 (3)	C15—C20—C19	120.5 (3)
C5—C6—H6	119.6	C15—C20—H20	119.8
C1—C6—H6	119.6	C19—C20—H20	119.8
C12—C7—C8	120.7 (3)	C22—C21—C26	120.2 (3)
C12—C7—N1	119.1 (3)	C22—C21—N3	118.5 (2)
C8—C7—N1	120.2 (3)	C26—C21—N3	121.1 (2)
C7—C8—C9	119.8 (3)	C21—C22—C23	121.1 (3)
C7—C8—H8	120.1	C21—C22—H22	119.4
C9—C8—H8	120.1	C23—C22—H22	119.4
C10—C9—C8	118.2 (3)	C22—C23—C24	117.8 (3)
C10—C9—C13	121.9 (3)	C22—C23—C27	120.6 (3)
C8—C9—C13	119.9 (3)	C24—C23—C27	121.5 (3)
C11—C10—C9	122.4 (3)	C25—C24—C23	122.4 (3)
C11—C10—H10	118.8	C25—C24—H24	118.8
C9—C10—H10	118.8	C23—C24—H24	118.8
C10—C11—C12	118.7 (3)	C24—C25—C26	118.5 (3)
C10—C11—C14	121.2 (3)	C24—C25—C28	121.0 (3)
C12—C11—C14	120.1 (3)	C26—C25—C28	120.4 (3)
C7—C12—C11	120.2 (3)	C21—C26—C25	120.0 (3)
C7—C12—H12	119.9	C21—C26—H26	120.0
C11—C12—H12	119.9	C25—C26—H26	120.0
C9—C13—H13A	109.5	C23—C27—H27A	109.5
C9—C13—H13B	109.5	C23—C27—H27B	109.5
H13A—C13—H13B	109.5	H27A—C27—H27B	109.5
C9—C13—H13C	109.5	C23—C27—H27C	109.5
H13A—C13—H13C	109.5	H27A—C27—H27C	109.5
H13B—C13—H13C	109.5	H27B—C27—H27C	109.5
C11—C14—H14A	109.5	C25—C28—H28A	109.5
C11—C14—H14B	109.5	C25—C28—H28B	109.5
H14A—C14—H14B	109.5	H28A—C28—H28B	109.5
C11—C14—H14C	109.5	C25—C28—H28C	109.5
H14A—C14—H14C	109.5	H28A—C28—H28C	109.5
H14B—C14—H14C	109.5	H28B—C28—H28C	109.5
			
O1—S1—N1—C7	−69.6 (2)	O5—S2—N3—C21	64.2 (3)
O2—S1—N1—C7	161.4 (2)	O6—S2—N3—C21	−166.2 (2)
C1—S1—N1—C7	44.2 (3)	C15—S2—N3—C21	−49.3 (3)
O1—S1—C1—C2	−168.8 (2)	O5—S2—C15—C20	−10.3 (2)
O2—S1—C1—C2	−40.6 (3)	O6—S2—C15—C20	−139.9 (2)
N1—S1—C1—C2	76.1 (3)	N3—S2—C15—C20	105.8 (2)
O1—S1—C1—C6	6.1 (3)	O5—S2—C15—C16	165.8 (2)
O2—S1—C1—C6	134.3 (2)	O6—S2—C15—C16	36.2 (3)
N1—S1—C1—C6	−109.0 (2)	N3—S2—C15—C16	−78.1 (2)
C6—C1—C2—C3	0.2 (4)	C20—C15—C16—C17	0.2 (4)
S1—C1—C2—C3	175.0 (2)	S2—C15—C16—C17	−175.8 (2)
C6—C1—C2—N2	179.6 (3)	C20—C15—C16—N4	−179.3 (2)
S1—C1—C2—N2	−5.6 (4)	S2—C15—C16—N4	4.7 (4)
O3—N2—C2—C1	−41.2 (4)	O8—N4—C16—C17	43.4 (4)
O4—N2—C2—C1	140.3 (3)	O7—N4—C16—C17	−132.8 (3)
O3—N2—C2—C3	138.3 (3)	O8—N4—C16—C15	−137.1 (3)
O4—N2—C2—C3	−40.3 (4)	O7—N4—C16—C15	46.7 (4)
C1—C2—C3—C4	1.0 (4)	C15—C16—C17—C18	−1.9 (4)
N2—C2—C3—C4	−178.4 (3)	N4—C16—C17—C18	177.6 (3)
C2—C3—C4—C5	−1.7 (5)	C16—C17—C18—C19	2.1 (5)
C3—C4—C5—C6	1.3 (5)	C17—C18—C19—C20	−0.6 (5)
C4—C5—C6—C1	0.0 (5)	C16—C15—C20—C19	1.4 (4)
C2—C1—C6—C5	−0.7 (4)	S2—C15—C20—C19	177.7 (2)
S1—C1—C6—C5	−175.9 (2)	C18—C19—C20—C15	−1.2 (5)
S1—N1—C7—C12	−131.0 (2)	S2—N3—C21—C22	128.6 (2)
S1—N1—C7—C8	51.7 (3)	S2—N3—C21—C26	−56.2 (3)
C12—C7—C8—C9	0.7 (4)	C26—C21—C22—C23	−0.6 (4)
N1—C7—C8—C9	178.0 (2)	N3—C21—C22—C23	174.6 (2)
C7—C8—C9—C10	−1.5 (4)	C21—C22—C23—C24	0.8 (4)
C7—C8—C9—C13	−179.3 (3)	C21—C22—C23—C27	−177.6 (3)
C8—C9—C10—C11	0.9 (5)	C22—C23—C24—C25	−0.5 (4)
C13—C9—C10—C11	178.7 (3)	C27—C23—C24—C25	177.9 (3)
C9—C10—C11—C12	0.4 (5)	C23—C24—C25—C26	−0.1 (4)
C9—C10—C11—C14	−179.2 (3)	C23—C24—C25—C28	−177.4 (3)
C8—C7—C12—C11	0.6 (4)	C22—C21—C26—C25	0.0 (4)
N1—C7—C12—C11	−176.7 (2)	N3—C21—C26—C25	−175.1 (2)
C10—C11—C12—C7	−1.2 (4)	C24—C25—C26—C21	0.3 (4)
C14—C11—C12—C7	178.4 (3)	C28—C25—C26—C21	177.7 (3)

### Hydrogen-bond geometry (Å, º)

**Table:**

*D*—H···*A*	*D*—H	H···*A*	*D*···*A*	*D*—H···*A*
N1—H1*N*···O1^i^	0.82 (2)	2.40 (2)	3.089 (3)	142 (3)
N1—H1*N*···O3	0.82 (2)	2.52 (3)	2.893 (4)	109 (2)
N3—H3*N*···O7	0.83 (2)	2.42 (3)	2.963 (3)	124 (3)
N3—H3*N*···O6^ii^	0.83 (2)	2.54 (2)	3.195 (3)	136 (3)

Symmetry codes: (i) −*x*, *y*−1/2, −*z*+1/2; (ii) −*x*+1, −*y*+1, −*z*.

## References

[bb1] Adsmond, D. A. & Grant, D. J. W. (2001). *J. Pharm. Sci.* **90**, 2058–2077. 10.1002/jps.1157 11745765

[bb2] Chaithanya, U., Foro, S. & Gowda, B. T. (2012). *Acta Cryst.* E**68**, o2627. 10.1107/S1600536812034009 PMC3435655 22969528

[bb3] Gowda, B. T. & Weiss, A. (1994). *Z. Naturforsch. Teil A*, **49**, 695–702.

[bb4] Oxford Diffraction (2009). *CrysAlis CCD* and *CrysAlis RED* Oxford Diffraction Ltd, Yarnton, England.

[bb5] Shahwar, D., Tahir, M. N., Chohan, M. M., Ahmad, N. & Raza, M. A. (2012). *Acta Cryst.* E**68**, o1160. 10.1107/S1600536812011658 PMC3344102 22606105

[bb6] Sheldrick, G. M. (2008). *Acta Cryst.* A**64**, 112–122. 10.1107/S0108767307043930 18156677

[bb7] Shetty, M. & Gowda, B. T. (2004). *Z. Naturforsch. Teil B*, **59**, 63–72.

[bb8] Spek, A. L. (2009). *Acta Cryst.* D**65**, 148–155. 10.1107/S090744490804362X PMC2631630 19171970

